# The PATHOGENesis of Food Allergy

**DOI:** 10.3389/fped.2019.00484

**Published:** 2019-11-21

**Authors:** Peck Y. Ong

**Affiliations:** ^1^Division of Clinical Immunology and Allergy, Children's Hospital Los Angeles, Los Angeles, CA, United States; ^2^Department of Pediatrics, Keck School of Medicine, University of Southern California, Los Angeles, CA, United States

**Keywords:** archaea, bacteria, skin, atopic dermatitis, cross-reactivity, egg allergy, IgE sensitization

## Summary

The current paper suggests that egg allergy may arise due to microbial proteins that are homologous to egg allergens. These microbial proteins elicit an allergic response and lead to the development of specific microbial IgE molecules. These molecules cross-react with egg allergens and result in egg allergy. Some examples of microbial proteins that share similar sequences as egg allergens are presented in this paper.

Food allergy has a negative impact on the quality of life and nutrition. In addition, it can lead to life-threatening reactions. The pathogenesis of food allergy is still not fully understood. Many young children develop food allergy with no known prior ingestion of the food allergen in question. The strong connection between food allergy and atopic dermatitis has been well-documented. While the prevalence of food allergy in the general pediatric population is 4–5%, the prevalence of food allergy in atopic dermatitis is at least 20% (1). This connection between food allergy and atopic dermatitis has led to the suggestion that the skin may be the site of food IgE sensitization, leading to food allergy. The dual-allergen-exposure hypothesis suggests that food allergens are sensitized via eczema, whereas early gastro-intestinal exposure leads to tolerance (2). This hypothesis is supported by multiple basic studies that provide evidence for IgE sensitization via the skin [reviewed in (3)]. In addition, it has been shown that environmental level of peanut allergens is increased in children who developed peanut allergy (4). The early introduction of peanut has led to the prevention of peanut allergy (5). Likewise, early introduction of egg has also met with some success, although the results were not as consistent (3). It has been suggested that improvement in eczema, in addition to early introduction of egg, is needed for successful prevention of egg allergy (6). This suggestion further highlights the importance of skin in the pathogenesis of food allergy. A hypothesis is proposed here that the interaction between the neonatal skin and microbial proteins is important for the development of IgE sensitization and egg allergy.

The current hypothesis predicts the presence of microbial proteins that are homologous to egg allergens. Table 1 shows the microbial proteins that share homology with the IgE-binding domains of Gal d 1 (ovomucoid) (7). Four out of the 6 microbial proteins share > 60 % identity with a clinically-relevant IgE-binding region of Gal d 1 (FNPVCGTDGVTYDN) (8). Significant homology was also found between microbial proteins and Gal d 2 (ovalbumin), but no homology was found in the IgE-binding domains of Gal d 2 (9) (data not shown). To further confirm the correlation between microbial proteins and Gal d 1, prospective studies may be carried out to look for these microbial pathogens in atopy-prone neonates and correlate with neonates who eventually develop allergy to Gal d 1. These microbial pathogens can also be inoculated in animal models to show the development of specific IgE that cross-react with Gal d 1. Atopy-prone neonates who are born to parent with atopic dermatitis, asthma or allergic rhinitis have inherent skin barrier defects that predispose them to develop atopic dermatitis. Microbial pathogens are capable of evading these barrier defects to interact with the cutaneous immune system in these children. The processing of microbial proteins by antigen-presenting cells and subsequent presentation of antigenic peptides to T helper type 2 cells leads to the production of IL-4 and IL-13, which induce B cells to express specific IgE molecules. Bacterial allergy has been described more than half a century ago (10). It is also known that specific IgE to staphylococcal toxins can develop in young children with atopic dermatitis (11). Bacteria such as bacteroides can be acquired during birth or they can be part of neonates' microbiome (12). The skin also contains a wide array of microbial pathogens that can participate in the development of allergy (13). More recent data suggests that many microbial organisms (e.g., proteobacteria and archaea) previously thought to exist only in the environment such as soil, fresh or marine waters are now found to be part of the human skin microbiome (14, 15). Proteobacteria and bacteroides are also known to be present in atopic dermatitis lesions (16). The current proposal is conceptual that microbial proteins can be a sensitizing source in the development of food allergy in predisposed children. Preliminary data also suggests the presence of homologous proteins between microbes and other food allergens including peanut and cow's milk (unpublished data). Whether these microbial pathogens have a direct interaction with neonates, leading to the development of specific IgE, remains to be proven. It is possible that the prevention of egg allergy requires a different approach by targeting microbial pathogens.

**Table 1:**
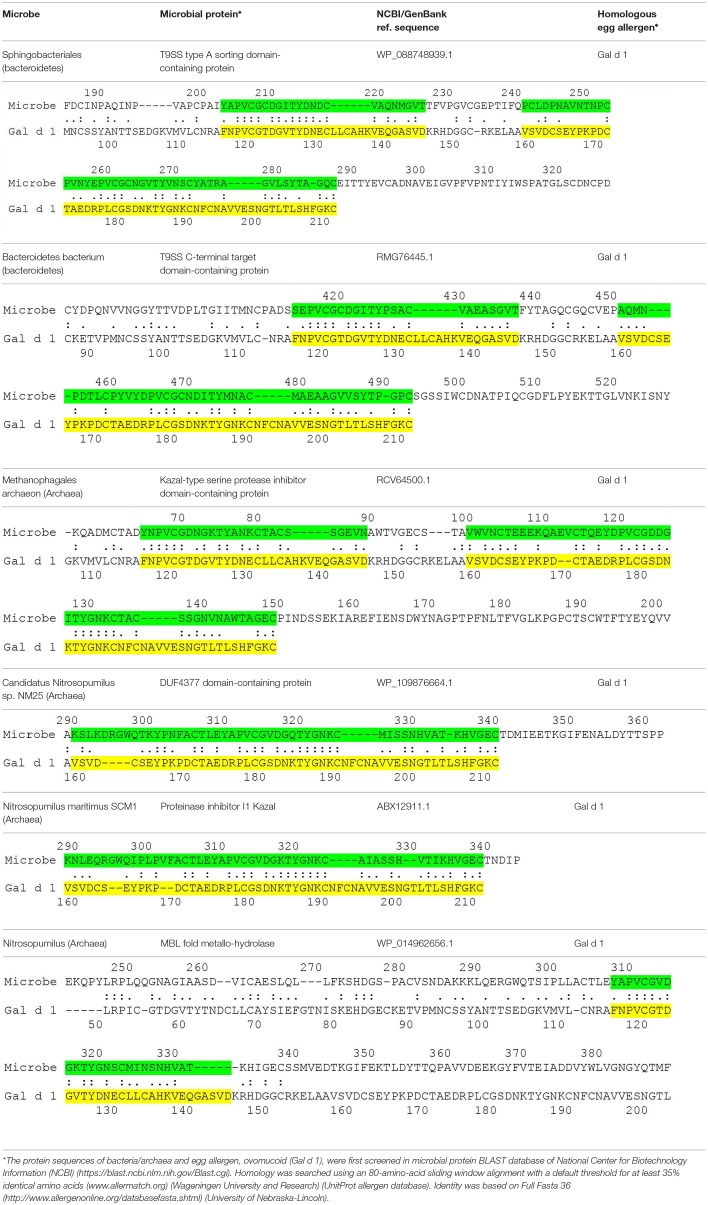
Microbial proteins that share similar sequences as the IgE-binding domains of Gal d 1.

## Author Contributions

PO conceived the idea, generated the data, and wrote the paper.

### Conflict of Interest

The author declares that the research was conducted in the absence of any commercial or financial relationships that could be construed as a potential conflict of interest.
